# Lower leptin levels in young non-obese male smokers than non-smokers

**DOI:** 10.1080/03009730902761631

**Published:** 2009-09-07

**Authors:** Bayram Koc, Fatih Bulucu, Nuri Karadurmus, Mustafa Şahin

**Affiliations:** ^1^Department of Internal Medicine, Gulhane School of MedicineEtlik 06018 AnkaraTurkey; ^2^Internal Medicine Department, Air Force HospitalEskisehirTurkey; ^3^Department of Endocrinology, Gulhane School of MedicineEtlik 06018, AnkaraTurkey

**Keywords:** Leptin, male, smoking

## Abstract

Since the effect of smoking on plasma leptin has been divergent in clinical trials, which might have occurred due to selection of heterogeneous study populations, we investigated whether there is such an association in a group of healthy, non-obese, young male adults.

A total of 54 smokers (mean age: 21.18±1.62; body mass index (BMI): 19.60±0.85) and 26 non-smokers (mean age 21.69±3.0; BMI: 21.59±1.39) with similar daily physical activities and diet and without any documented disease were enrolled, and their plasma leptin levels were determined for the comparison between the two groups.

The mean BMI and plasma leptin of smokers were significantly lower than in non-smokers. Plasma leptin in the smokers group correlated inversely with BMI and the amount of daily smoking. Below BMI 20 kg/m^2^ and between 20.0 and 20.9 kg/m^2^ the plasma leptin levels in smokers were significantly lower when compared to non-smokers.

Plasma leptin is decreased in healthy, young non-obese male smokers independently of the amount of body fat. High amount of smoking is associated with lower serum leptin as well.

## Introduction

Long-time smoking is characterized by reduced body-weight (1–3), while cessation of smoking usually leads to rapid weight-gain (4–7). The probable causes of weight-gain are increased food intake, decreased resting metabolic rate and physical activity, and increased lipoprotein lipase activity (7). Moreover, weight concerns affect the motivation to quit smoking (8). However, the molecular factors regulating food intake and body-weight in smokers have not yet been clearly identified. Leptin, a polypeptide hormone secreted by adipocytes, has been shown to influence body-weight mainly by reducing food intake and increasing energy expenditure through central and peripheral mechanisms (9–11). Leptin has a diurnal rhythm and a positive correlation with the degree of adiposity, body mass index (BMI), and circulating insulin levels (12–15). Gender differences also determine the plasma leptin levels (16).

Since there is a close association of body-weight with smoking and serum leptin, many authors have investigated the interaction between leptin and smoking. However, the effect of smoking on plasma leptin has been divergent in clinical trials. Some studies have mentioned decreased serum leptin in smokers (17–23) while others found no difference between smokers and non-smokers regarding circulating leptin (24–26). Since these controversial results might have occurred due to selection of study populations in those studies, which frequently included heterogeneous groups of individuals regarding age, gender, and BMI, we investigated the association between plasma leptin levels and smoking in a homogenous group of healthy, non-obese, young male adults with appropriate controls.

## Methods

### Study population

The study was conducted on 80 young, non-obese male adults (mean age: 21.35±2.16 years; BMI: 20.25±1.41) who were allocated into two groups according to their smoking habits as 54 smokers (mean age: 21.18±1.62; BMI: 19.60±0.85) and 26 non-smokers (mean age 21.69±3.0; BMI: 21.59±1.39). Being a smoker was defined as smoking cigarettes for at least 2 years, with more than 5 cigarettes daily. Only a ‘never-smoker’ was accepted as a non-smoker. No ex-smoker was included in any group. The participants of the study were the soldiers from a troop and had similar daily physical activities and diet for at least 6 months. None of the participants had any documented disease nor were taking any medication, including over-the-counter drugs. Physical activity levels and glucose tolerance status were similar for smokers and non-smokers.

The study was approved by the Ethics Committee of Gulhane Medical School. All subjects gave written informed consent before entrance in the study.

According to the daily numbers of cigarettes, the patients were grouped as heavy smokers (*n*=12) (more than 20 cigarettes a day), moderate smokers (*n*=12) (between 10 and 20 cigarettes a day), and mild smokers (*n*=30) (fewer than 10 cigarettes a day).

### Anthropometric measurements

Height and weight were determined with the participant wearing underpants, without shoes. BMI was calculated as weight (kg)/height (m)^2^.

### Analyses

Overnight fasting venous blood samples were collected at 08.00 for analysis of leptin in cold test tubes containing 0.084 mL Ethylene Diamine Tetraacetic Acid (EDTA) (0.34 mol/L). Leptin was measured by a radioimmunoassay using Human Leptin IRMA (DSL-23100 Leptin-Coated Tube Immunoradiometric Assay Kit) (Diagnostic Systems Laboratories Inc., Texas, USA). Serum was stored at -30°C until analyses, and all measurements were performed in duplicate.

### Statistical analyses

All data are presented as mean±SD unless otherwise noted. Student's *t* test was used to compare the parameters of the two groups. The associations between serum leptin levels and BMI or daily cigarette number were investigated by Spearman's correlation analysis.

## Results

**Figure 1.**
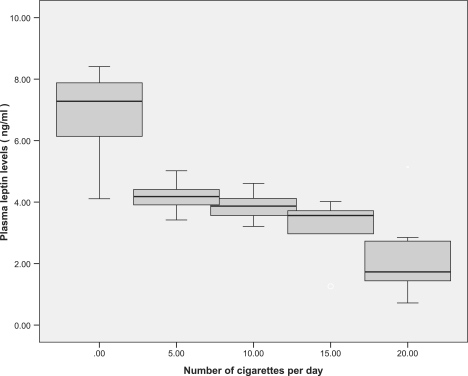
Plasma leptin levels according to number of cigarettes per day.

Age, blood pressure, total cholesterol, triglyceride, and fasting glucose levels were all similar in both the smoker and non-smoker groups. The mean BMI of smokers was lower than non-smokers (19.60±0.85 kg/m^2^ versus 21.59±1.39 kg/m^2^) (*P <*0.001). The plasma leptin levels in the smoker group were significantly lower than in non-smokers (3.47±1.1 ng/mL versus 6.63±1.5 ng/mL) (*P =*0.006). Plasma leptin in the smokers group correlated inversely with BMI (*r*=0.83, *P <*0.001) and the amount of daily smoking (*r =*-0.77, *P <*0.001) (Figure 1).

The smokers and non-smokers were subanalyzed according to BMI values. Below 20 kg/m^2^ (*n*=22; 12 smokers, 10 non-smokers) and between 20.0 and 20.9 kg/m^2^ (*n*=30; 18 smokers, 12 non-smokers) of BMIs, the plasma leptin levels in smokers were significantly lower (*P =*0.009 and *P =*0.024, respectively) when compared to non-smokers with the same BMI values. Since there were only two individuals in the smoker group who had a BMI above 21, comparison of plasma leptin levels in the subjects having BMIs higher than this value was not performed.

## Discussion

The present study, which was designed to search for any relation between smoking and plasma leptin levels in healthy and young non-obese males, indicates that smokers have significantly lower plasma leptin levels. Low leptin levels in smokers compared to non-smokers within the similar BMI interval suggest that smoking is associated with low plasma leptin independently of the body fat content. Moreover, there was an inverse association between the number of cigarettes smoked per day and the level of plasma leptin.

Leptin, which plays an important role in body-weight homeostasis through reducing food intake and increasing thermogenesis, has been studied in smokers widely. While a number of studies indicated that smoking does not affect plasma leptin levels (12,24–26), some others reported lower plasma leptin levels in smokers (17,18,20–23). More controversies exist considering the reports with increased plasma leptin in smoking people (27,28). Detailed analyses of these studies indicate selection of study population as the most probable reason for these conflicting results. For instance, Larsson et al. found no relation between smoking habits and plasma leptin levels in their study with postmenopausal, Caucasian, non-obese smoking women (26). Similar results were also reported in type 2 diabetic smoking Japanese males (25). On the other hand, Hodge et al. in a group of smokers in the South Pacific Islands (17) and Wei et al. in non-Hispanic whites and Mexican Americans (18) measured low plasma leptin levels. Though these results led to a question of an ethnicity-based association between plasma leptin levels and smoking, Donahue et al. reported in their large multiethnic study that ethnicity did not establish a direct relation between plasma leptin and life habits (20). According to their study, cigarette smoking was associated with lower plasma leptin levels in both men and women.

Another possible mechanism for the lower leptin in smokers could be related to the fact that smoking increases plasma catecholamines and free fatty acid concentrations, and lipolysis which may decrease leptin concentrations (29–32). Also, the nicotinic effect of smoking may alter the sensitivity of hypothalamic leptin receptors and modulate leptin synthesis and a reduction in body-weight (16,31,32).

The discrepant results could also be explained by other factors such as diet, exercise and other lifestyle habits, hormones, and inflammatory markers which may also regulate leptin secretion (29).

In a recent study, leptin levels were significantly higher in women compared with men, and for both sexes were positively correlated with both BMI and the well studied marker of inflammation Creactive protein (CRP) (33).

The link between smoking and insulin resistance syndrome was well identified (16,33–35). In addition, smoking can increase the release of catecholamines, cortisol, adrenocorticotrophic hormone, and growth hormone, and alter the sympathetic system activity (29,34). These effects may be responsible for the decrement of insulin-mediated glucose uptake in smokers (36,37). Correlation between fasting insulin and leptin levels was also previously reported (38). Thus, insulin resistance is likely to be associated with regulation of leptin metabolism in smokers. On the other hand, multiple regressions analysis of the study of Donahue et al. revealed that the effect of smoking on plasma leptin levels seems to be independent of fasting insulin levels (20). Since no overweight individual was included neither in the study nor the control group, and all subjects were young and fairly well exercised, we did not measure fasting plasma insulin levels.

In conclusion, the present study indicates that plasma leptin is decreased in healthy, young non-obese male smokers independently of the amount of body fat, and high amount of smoking is associated with lower serum leptin. Thus, smoking appears to be among the direct modulators of leptin metabolism.
